# The Medical Defence Union and the London and Counties Medical Protection Society (Limited)

**Published:** 1893-12-09

**Authors:** W. Bruce Clarke, G. A. Heron, Hugh Woods

**Affiliations:** Amalgamation Sub-Committee, London and Counties Medical Protection Society. Harley Street, W.; Amalgamation Sub-Committee, London and Counties Medical Protection Society. Harley Street, W.; Amalgamation Sub-Committee, London and Counties Medical Protection Society. Harley Street, W.


Sir,—In your last week's issue you publish a document in which the council of the Medical Defence Union profess to give a *résumé* of certain negotiations, which have recently been carried on in the hope that they might lead to the amalgamation of the London and Counties Medical Protection Society with the Medical Defence Union. These negotiations were conducted by two committees appointed for the purpose, one from each Socieey.

That a *résumé* of such negotiations should be published in the medical papers in due time was a course to which no one could take exception, and was, indeed, assented to by both parties to the conference.

But this *résumé* is misleading, because it is an illustration of what is an undoubted suppression of facts; and a knowledge of these suppressed facts is essential to anyone who would fairly judge between the two Societies, and decide which of the two should bear the blame of the breakdown of the negotiations for amalgamation. In the fourth paragraph from the end of the *résumé* in question, this statement is made:

"In view, however, of the fact that the London and Counties Medical Protection Society contemplated winding up," &c.

Now it was well known to every member of the two Special Committees that the Medical Protection Society had never been asked to wind up. It is undoubtedly true that, in our wish to foster, in every possible way, whatever might lead to the formation of one large and powerful society for the protection of medical men and their interests, we, the sub-committee of the Medical Protection Society, had undertaken to suggest to our council that they should consent to the winding up of our affairs, after the amalgamation had beon practically completed; but until this *résumé* was published, our council had never even heard of this suggestion of ours. This is an accurate statement of our position, a very different one from that which is contained in the *résumé* which was drawn up by men who, having a perfect knowledge of the facts of the case, could yet bring themselves to state that the London and Counties Medical Protection Society "contemplated winding up."

But in addition to issuing misleading statements, those who drew up and sanctioned the publication of the *résumé* in question have deliberately suppressed a fact which is of the first importance to the understanding of the points at issue. For although the *résumé* correctly states that the Defence Union demanded that a list of the nominees of the London and Counties Medical Protection Society should be furnished them, it ingeniously omits to state that we, the sub-committee, whilst very strongly objecting to any such proposal, made the following counter suggestion:

"The Committee of the Protection Society, whilst objecting to the principle of scrutiny of members of Council, and in view of the proposition made by the Council of the Medical Defence Union, requires that the Medical Defence Union shall submit their list of Councillors for the proposed joint Society to the same measure of criticism to which the Medical Defence Union intends to submit the list of Councillors to be proposed by the Medical Protection Society."

But until Tuesday, November 28th, on which day the *résumé* was in type for publication, we had received no copy of it, and had no opportunity of knowing whether the Defence Union would grant the right of scrutiny to our society, which it was determined to exact for itself, and until this was known it was useless to lay its proposals before our General Committee.

We expressly stated, on more than one occasion, to the Amalgamation Committee of the Medical Defence Union, that we were merely the delegates of our society, that we had no power to finally decide anything; and that when we had learned how far the Medical Defence Union would meet us in amalgamation we would carry its proposals to our committee for ratification or refusal. That opportunity was never offered us; and the statement which appeared in your issue of December 2nd reached us, as we have said, so late that it barely afforded time to our Secretary to pen a hasty reply without consultation with his other two colleagues, Dr. Heron and Mr. Bruce Clarke.

Our desire for union was based on the sincere belief that union in the medical profession, as elsewhere, means strength, and that one Society was better than two when the objects of both are so nearly identical. The cause which led to the formation of a second Society had, we had every reason to hope, ceased to exist. It is a matter of sincere regret to us that our efforts in the direction of amalgamation have been frustrated by the publication of this untimely *ex parte résumé*, and that no opportunity was afforded us or our General Committee for its discussion. At the same time we cordially agree with Mr. Victor Horsley's words, "It is only due to the profession that an account" (of course he means a correct account) "of the negotiations should be published."—We are, sir, yours odediently,

W. Bruce Clarke,
G. A. Heron,
Hugh Woods,
Amalgamation Sub-Committee, London and Counties Medical Protection Society.

Harley Street, W., December 5th, 1893.

